# STAKEHOLDER INSIGHTS: STANDARDIZING END-OF-LIFE CARE TRANSITIONS FOR NURSING HOME RESIDENTS WITH DEMENTIA

**DOI:** 10.1093/geroni/igad104.3601

**Published:** 2023-12-21

**Authors:** Susanny Beltran, Latarsha Chisholm, Emily Jaijairam

**Affiliations:** University of Central Florida, Orlando, Florida, United States; University of Central Florida, Orlando, Florida, United States; University of Central Florida, Orlando, Florida, United States

## Abstract

Sixty-seven percent of dementia patients die in nursing homes (NHs), where end-of-life care is often suboptimal, and hospice not accessed in a timely manner. The eligibility criteria for the Medicare Hospice Benefit (< 6 months prognosis) poses challenges for patients with Alzheimer’s disease and related dementias given the variable disease progressions that characterize most dementias. This study describes current NH processes used to identify dementia residents to refer to hospice, and stakeholders’ input into the design of a standardized process for clinical decision-making, using the Mitchell Index, a dementia mortality prediction tool. Individual interviews and focus groups were conducted with NH administrators, medical directors, directors of nursing, MDS coordinators, and social workers (N=11). Participants completed an online survey including facility (e.g., size, profit status) and individual demographics (e.g., years of experience), then participated in an interview that involved a brief presentation of the proposed standardized process using the Mitchell Index. Qualitative data analysis followed a deductive approach, guided by constructs from the Consolidated Framework for Implementation Research (CFIR). Preliminary analyses indicate providers believe the Mitchell Index can be successfully adapted for NH use. Participants stated the tool added something to current processes (CFIR relative advantage), would be easy to use (CFIR complexity), and pointed to external influences (i.e., regulations; CFIR domain 2: outer setting) which would be important to consult in later stages of intervention development. Overall, nursing home stakeholders endorse the potential for a standardized process using the Mitchell Index to be acceptable and feasible in high-Medicaid nursing homes.

